# Transesophageal Echocardiogram Before Cardioversion in Atrial Fibrillation Patients

**DOI:** 10.7759/cureus.39702

**Published:** 2023-05-30

**Authors:** Victor O Adedara, Vagisha Sharma, Hassan Nawaz, Jonathan Reyes-Rivera, Sumera Afzal-Tohid, Patel T Pareshbhai, Sri P Boyapati, Alireza Sharafshah

**Affiliations:** 1 Medicine, St. George's University School of Medicine, St. George’s, GRD; 2 Medicine, Vardhman Mahavir Medical College and Safdarjung Hospital, New Delhi, IND; 3 Medicine, Nishtar Medical University and Hospital, Multan, PAK; 4 Medicine, Universidad Autonoma de San Luis Potosi, San Luis Potosi, MEX; 5 Medicine, Ziauddin University, Karachi, PAK; 6 Medicine, S.S. (Sri Sayaji) Hospital, Petlad, IND; 7 Medicine, Siddartha Medical College, Dr. YSR University of Health Sciences, Vijayawada, IND; 8 Genetics, IMG Helping Hands, Newark, USA

**Keywords:** thromboembolic event, cardiology research, cardioversion, atrial fibrillation, transesophageal echocardiography

## Abstract

Transesophageal echocardiography (TEE) offers an invaluable, non-invasive avenue for diagnosing and managing various cardiac conditions, including atrial fibrillation (AF). As the most common cardiac arrhythmia, AF affects millions and can lead to severe complications. Cardioversion, a procedure to restore the heart's normal rhythm, is frequently conducted on AF patients resistant to medication. Due to inconclusive data, TEE's utility prior to cardioversion in AF patients remains ambiguous. Understanding TEE's potential benefits and limitations in this population could significantly influence clinical practice. This review aims to scrutinize the current literature on the use of TEE before cardioversion in AF patients. The principal objective is to understand TEE's potential benefits and limitations comprehensively. The study seeks to offer a clear understanding and practical recommendations for clinical practice, thereby improving the management of AF patients before cardioversion using TEE.

A literature search of databases was conducted using the keywords "Atrial Fibrillation," "Cardioversion" and "Transesophageal echocardiography," resulting in 640 articles. These were narrowed to 103 following title and abstract reviews. After applying exclusion and inclusion criteria with a quality assessment, 20 papers were included: seven retrospective studies, 12 prospective observational studies, and one randomized controlled trial (RCT). Stroke risk associated with direct-current cardioversion (DCC) potentially results from post-cardioversion atrial stunning. Thromboembolic events occur post cardioversion, with or without prior atrial thrombus or cardioversion complications. Generally, cardiac thrombus localizes in the left atrial appendage (LAA), a clear contraindication to cardioversion. Atrial sludge without LAA thrombus in TEE is a relative contraindication. TEE before electrical cardioversion (ECV) in anticoagulated AF individuals is uncommon. In AF patients planned for cardioversion, contrast enhancement facilitates thrombus exclusion in TEE images, reducing embolic events. Left atrial thrombus (LAT) frequently occurs in AF patients, necessitating TEE examination. Despite the increased use of pre-cardioversion TEE, thromboembolic events persist. Notably, patients with post-DCC thromboembolic events had no LA thrombus or LAA sludge. The use of TEE-guided DCC has grown due to its ability to detect atrial thrombi pre-cardioversion, aiding risk stratification. Thrombus in the left atrium also signals an elevated risk of future thromboembolic events in AF patients. While atrial stunning post cardioversion detected by TEE is a significant risk factor for future thromboembolic events, further evidence is required. Therapeutic anticoagulation is essential during and post cardioversion, even if no atrial thrombus is detected. Current data recommends cardioversion guided by TEE, particularly in outpatient settings.

## Introduction and background

Transesophageal echocardiography (TEE) is a non-invasive diagnostic tool that allows for detailed visualization of the heart and its structures using ultrasound waves. Physicians utilize TEE the most when they try to find more details than a standard echocardiogram. It is a widely accepted and commonly used technique in diagnosing and managing various cardiac conditions, including atrial fibrillation (AF) [1]. According to the American Heart Association, AF is the most common cardiac arrhythmia, affecting an estimated 2.7-6.1 million individuals in the United States alone [2]. It is characterized by an irregular and often rapid heart rate, which can lead to severe complications such as stroke, heart failure, and other cardiac issues. Exclusion of the thrombus is extremely important with respect to the planned reversal of sinus rhythm [3].

Cardioversion is a procedure that aims to restore the normal rhythm of the heart and is often performed in AF patients who are resistant to medical management [4]. However, the use of TEE before cardioversion in AF patients still needs to be better understood. Currently, data on routine TEE before cardioversion is inconclusive [3]. The motivation for this review is to study the current literature on using TEE as a diagnostic tool before cardioversion in AF patients. The study aims to comprehensively understand TEE's potential benefits and limitations in this population and guide clinical practice.

Patients with AF and flutter routinely require TEE with cardioversion [5]. The focus of this review is the current literature on the use of TEE before cardioversion in AF patients, with the research question being the role of TEE in the management of AF patients before cardioversion. The paper has been structured by reviewing the current literature on the topic, analyzing the findings, and discussing the implications for clinical practice. This review provides a comprehensive understanding of the potential benefits and limitations of this diagnostic tool and also provides practical recommendations for clinical practice. The importance of this review lies in the growing use of TEE in the management of AF patients and the need for further research to understand its potential benefits and limitations in this population.

## Review

Methods

**Figure 1:**
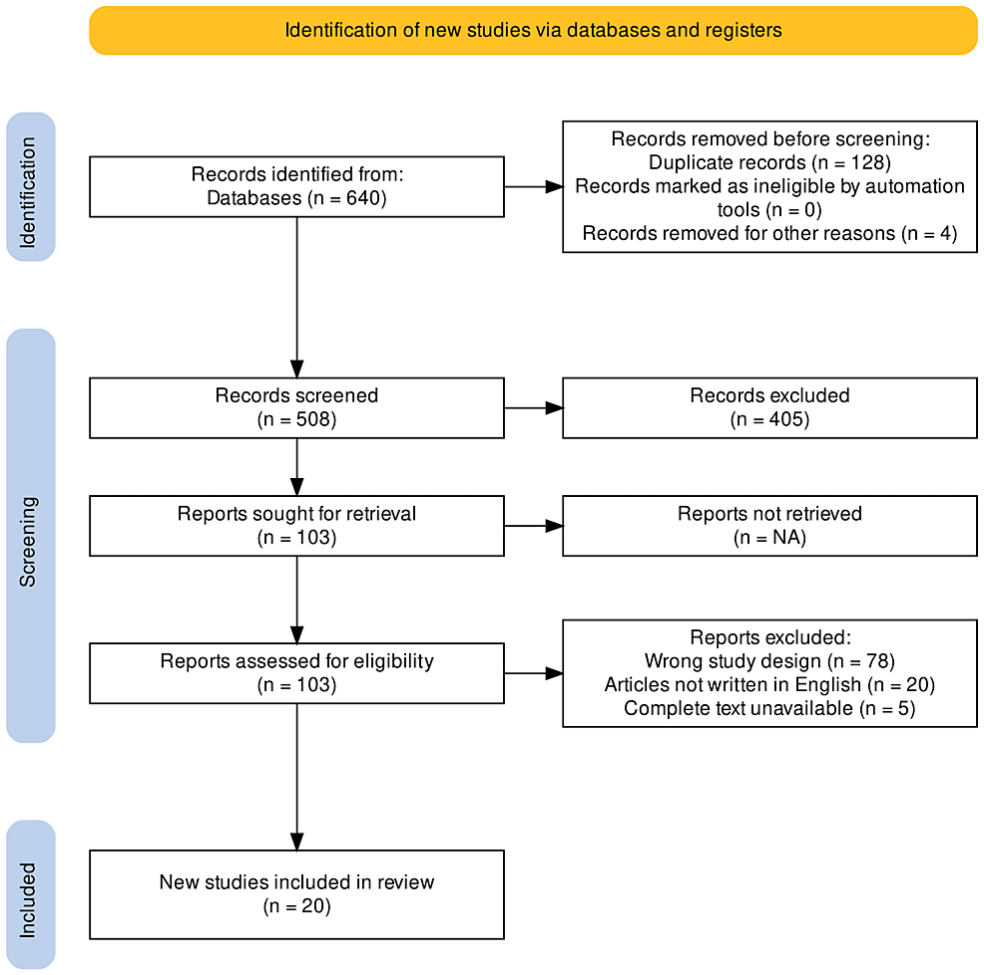
PRISMA schematic for this systemic review PRISMA: Preferred Reporting Items for Systematic Reviews and Meta-Analyses

A thorough medical literature search of various databases was done using the keywords "Atrial Fibrillation," "Cardioversion", and "Transesophageal echocardiography." A total of 640 results were shortlisted. A review of the article's title and abstract was done, after which 103 articles were selected. Further inclusion and exclusion criteria were implemented to refine the search, and a quality assessment was done. Ultimately 20 papers were included in this review article. The inclusion criteria comprised original studies, observational studies, and clinical trials. The exclusion criteria included narrative reviews, editorials, brief communications, case reports, case series, review articles, articles not written in English, and articles for which the complete text was unavailable. Seven of the 20 studies included were retrospective observational studies, while 12 were prospective observational studies (Figure 1). There was one randomized control trial (RCT) included

Result

There’s a risk of stroke associated with direct-current cardioversion (DCC), which might result from atrial stunning taking place post cardioversion. Thromboembolic events have been noted post cardioversion, irrespective of whether there was a prior thrombus in the atria or any complication during cardioversion. However, the cardiac thrombus, when present, was usually localized in the left atrial appendage (LAA) and is a well-recognized clear contra-indication to cardioversion. However, atrial sludge without LAA thrombus in TEE is a relative contra-indication to cardioversion. In anticoagulated individuals, TEE before electrical cardioversion (ECV) in AF is not commonly conducted [5]. In patients with AF planned for cardioversion, contrast enhancement is helpful since it makes excluding atrial thrombi by the TEE images easier and thus reduces the rate of embolic adverse events.

Left atrial thrombus (LAT) is not an uncommon finding in AF patients before cardioversion. Neither clinical nor routine two-dimensional (2D) echo examinations reliably identify LAT, so the current practice of TEE examination is needed. Over the past 20 years, the practice of TEE before cardioversion has undoubtedly increased, but the rate of thromboembolic events has not decreased to zero. The patients with thromboembolic events post-DCC had no evidence of LAT or LAA sludge. Table 1 summarizes the studies on AF patients with lone AF (LAF) and TEE examination.

**Table 1:** A brief review based on AF patients with LAF and TEE examination. LAT: left atrial thrombus; TEE: transesophageal echocardiography; DCC: direct current cardioversion; AF: atrial fibrillation; LVEF: left ventricular ejection fraction; LAF: lone atrial fibrillation; RCT: randomized control trial; SEC: spontaneous echo contrast; LA: left atrial; LAAEV: left atrial appendage emptying velocity; NYHA: New York Heart Association; CHA2DS2-VASc: congestive heart failure, hypertension, age ≥75 (doubled), diabetes, stroke (doubled), vascular disease, age 65 to 74 and sex category (female); NOAC: novel oral anticoagulants; LGE: late gadolinium enhancement

Author Name, Year	Study design	Study Population, Sample Size	Mean Age n sex of patients	Cardiac Pathology	Prevalence of LAT or sludge	Other Findings	Limitations	
Yarmohammadi et al., 2012 [6]	Retrospective study	2,705 patients	mean age, 66 ± 13 years; 68% men	Atrial fibrillation or atrial flutter	8% overall; P = .12	TEE-guided DCC was observed over the past 10 years (25% in 1999, 34% in 2008). TEE-guided DCC was also performed more often in the outpatient setting (21% in 1999, 37% in 2008)		
Fatkin et al., 1994 [7]	Prospective study	66 patients	Mean age 61 years; 62% men	All with AF, eight patients with mitral valve disease, and 58 patients with nonvalvular AF	Just one patient with LAT (1.4% overall)	Four patients had embolic events after cardioversion and none of them had evidence of LAT before cardioversion		
Black et al., 1993 [8]	Prospective study	40 patients	N/A	33 non-anticoagulated patients with nonvalvular AF, seven with atrial flutter	TEE detected Left atrial appendage thrombi in 5 pts (12%, p=0.03) and cardioversion was canceled in them.	Cerebral embolism occurred in 1 pt w/ AFib and left ventricular dysfunction, but no thrombus was detected by TEE before cardioversion.		
Klein et al., 1997 [9]	Prospective study	126 patients	N/A	AF longer than two days	TEE randomly done in 56 patients, LAT detected in seven (13%)			
Manning et al., 1993 [10]	Prospective study	94 patients	Mean age 72 ±13 years	AF longer than 2 days without long-term anticoagulation	TEE identified 12 patients with LAT (13%)	78 of 82 patients without thrombi underwent successful cardioversion, all without long-term anticoagulation, none of these patients had an embolic event.		
Smith et al., 2012 [11]	Retrospective review	2,999 patients	Mean age 59 ± 11	AF who had undergone TEE before cardioversion or RFA	263 eligible patients, two (0.8%; 95%CI, 0.21-2.7%) had thrombi on subsequent TEE			
Jung et al., 2013 [12]	RCT	180 patients with AF; 90 were examined with native imaging and contrast enhancement within the same examination (group 1), and 90 were examined with native TEE alone and served as control (group 2)	51 females; 65.2±13 years		In group 1, atrial thrombi were diagnosed in 14 (15.6%) during native and in 10 (11.1%) patients during contrast-enhanced imaging (p<0.001).	Of the 10 patients with thrombi in the contrast TEE group, 7 revealed a decreased LAA-flow (≤0,3m/s), and 8 showed moderate or marked SEC. Uncertain results were significantly more common during native imaging than with contrast-enhanced TEE (16 vs. 5 patients, p<0.01).	This study investigated the influence of contrast enhancement on interpretability of TEE for the detection of LAT compared to conventional TEE and assessed whether there are differences in the rate of thromboembolic events after electrical cardioversion.	
Malik at al., 2014 [13]	Retrospective study	600 consecutive patients with AF undergoing TEE prior to cardioversion	No thrombus group, thrombus group 65.6 ± 12.2 66.3 ± 12.9	AF undergoing TEE prior to cardioversion for the detection of LAT were analyzed	TEE identified LAT in 70 (11.6%) and dense (LA) SEC in 156 (26%).	A prior myocardial infarction; hypertension; CHADS(2) ≥ 2, prevalence was higher in patients with LAT. Patients with LAT had lower ejection fraction; higher LA diameter; dense LA SEC; and low LA appendage emptying velocity	This study includes those limitations that are inherent to any retrospective study. One of the limitations of the study was that it was a single-center trial. In this study, we did not evaluate TEE findings in conjunction with clinical outcomes.	
Yarmohammadi et al., 2014 [14]	Retrospective study	340 patients	Mean age, 66 ± 12 years; 75% men	UnderwentTEE to exclude LAA thrombus before electrical cardioversion or radiofrequency pulmonary vein isolation for AF	LAA sludge was independently predicted by enlarged LA area (odds ratio, 4.54; 95% confidence interval [CI], 2.38-8.67; P < .001), reduced LAA emptying velocity (odds ratio, 12.7; 95%CI, 6.11-26.44; P < .001), and reduced LVEF (odds ratio, 2.11; 95%CI, 1.03-4.32; P < .001).	Thromboembolic event and all-cause mortality rates in patients with sludge were 23% and 57%, respectively.		
Melduni et al., 2015 [15]	Prospective study	3,251 consecutive patients	Mean (SD) age was 69 (12.6) years; 67% were men	Patients with sustained AF undergoing first-time successful TEE-guided electrical cardioversion	Decreased probability of event-free survival with decreasing quartiles of LAAEV. Five-year cumulative event rates across first-fourth quartiles were 83%, 80%, 73%, and 73% (P < .001) for first AF recurrence; 7.5%, 7.0%, 4.1%, and 4.0%, for stroke (P = .01); and 31.3%, 26.1%, 24.1%, and 19.4%, for mortality (P < .001), respectively.	Patients with decreased LAAEV have an increased risk of AF recurrence, stroke, and mortality after successful electrical cardioversion. Real-time measurement of LAAEV by TEE may be a useful physiological biomarker for individualizing treatment decisions in patients with AF.		
Black et at., 1994 [16]	Retrospective study	17 patients		All had nonvalvular AF	TEE before cardioversion showed left atrial SEC in 5 patients and did not show atrial thrombus in any patient	All patients had embolic events after TEE-guided electrical/pharmaceutical cardioversion: 13 cerebral embolic events, and 4 peripheral embolism. None of them were anticoagulated at the time of embolic events. Findings suggest de novo atrial thrombosis after cardioversion or imperfect sensitivity of TEE for atrial thrombi and suggest that screening by TEE does not obviate the requirement for anticoagulant therapy at the time of and after cardioversion		
Antonielli et al., 1995 [17]	Prospective	74 patients		AF	46 without thrombus or prethrombotic conditions did not receive anticoagulation, 28 were followed with warfarin. 4 patients with LAT. Cerebral embolism occurred after cardioversion in one patient who did not receive anticoagulation	In AF candidates for cardioversion, exclusion of thrombi or prethrombotic conditions by TEE does not exclude the risk of thromboembolic events and the need for anticoagulant therapy	LAA function can be stunned or impaired immediately after cardioversion, favoring a thrombogenic milieu and subsequent embolic events. Therapeutic anticoagulation at the time of as well as after cardioversion is actually recommended	
Manning et al., 1995 [18]	Prospective	230 patients	Mean age 73 years; 105 men and 128 women	AF	34 patients had thrombi.	18 patients underwent cardioversion after prolonged anticoagulation 186 patients of 196 without thrombi had successful cardioversion without prolonged anticoagulation and none experienced a clinical thromboembolic event.		
Stoddard et al., 1995 [19]	Prospective	206 patients		AF	37 patients with LAT (18%)	In seven (41%) of 17 patients, new LAA SEC developed immediately after electric cardioversion. In one patient a LAA thrombus formed after electric cardioversion.	Cardioversion was safely done without or with < 7 days of anticoagulation prophylaxis in selected pts, but the potential for LAT to form after electric cardioversion makes anticoagulation advisable in all pts. The conventional recommendation of 3-4 weeks of anticoagulation prophylaxis before cardioversion is usually inadequate for LAT to resolve or to become immobile.	
Poterucha et al., 2016 [20]	Retrospective study	890 patients		Patients after the Fontan operation who underwent TEE-guided electrical cardioversion. Thirty-six patients (20 males, median age 29 (12-51)) underwent TEE-guided cardioversion of atrial arrhythmias (atrial flutter/intra-atrial reentrant tachycardia (75%); AF (25%)).	No embolic events occurred following cardioversion	After the Fontan operation, cardioversion of atrial arrhythmias improves ventricular function, atrioventricular valve regurgitation grade, and NYHA class.		
Akoum et al., 2013 [21]	Prospective	178 patients		AF, undergoing TEE and LGE-MRI prior to ablation or cardioversion	LAA thrombus was found in 12 patients (6.7%) while SEC was identified in 19 patients (10.7%)	Patients with thrombus had higher AF compared to patients without thrombus (26.9 ± 17.4% vs 16.7 ± 10.5%; P < 0.01). AF was also higher in patients with SEC (23.3 ± 13.7%) compared to those without SEC (16.7 ± 10.8%; P = 0.01). Patients with high AF (>20%) were more likely to have an LAA thrombus (odds ratio 4.6; P = 0.02) and SEC (odds ratio 2.6; P = 0.06)		
Squara et al., 2022 [22]	Prospective analysis	21 patients	76 (70–81) years; 48% males	Patients demonstrating atrial sludge without LAA thrombus in TEE and undergoing DCC for persistent AF	During the follow-up period of one month after DCC, no clinical embolic event, cardiac event, or unscheduled consultations/hospitalizations occurred. At one month, 67% of the patients remained in sinus rhythm.	No clinical event occurred in patients demonstrating atrial sludge without thrombus and undergoing DCC for AF.	No cerebral imaging was performed, safety of DCC was only assessed clinically. Accordingly, we cannot exclude the occurrence of silent microembolisms. study was non-comparative -cohort is quite small with 21 -limited the follow-up at 1 month	
Barysienė et al., 2022 [23]	Retrospective study	432 patients	Mean 65.0 ±11.5); 277 (64.1%) males	Patients who had been anticoagulated by means of oral anticoagulants (OACs) prior to planned cardioversion	TEE revealed LAT in seven patients. In warfarin and NOACs groups thrombi were revealed in five and two patients, respectively. TEE did not reveal any thrombi in patients with normal LV function; however, thrombi were found in two (6.1%) patients with slightly decreased LV function, and in five (17.9%) patients with markedly decreased LV function.	The risk of LAT in patients prepared for scheduled cardioversion in line with the guidelines is low. Higher risk of thrombi was present in patients with decreased LVEF (≤40%), CHA2DS2-VASc ≥5. In patients with decreased LVEF thrombi in LA were found more frequently than in patients with normal and slightly decreased LVEF (17.9% vs 2.2%, p=0.008).	TEE complications during 30 days after discharge were assessed.	
Feickert et al., 2020 [24]	Prospected analysis	403 (262 (65%) had no anticoagulation, 47 (11.7%) were on novel oral anticoagulant (rivaroxaban), 74 (18.4%) on warfarin INR>2, and 20 (5.0%) on warfarin INR<2)	69.6±10.3 years	Patients referred for electric cardioversion (anticoagulated and non-anticoagulated), undergoing TEE	In 41 (10.1%) there was LAT and in 154 (38.2%) SEC. Patients with LAT had a significantly lower LVEF% (p=0.001). Patients with SEC were significantly older (p=0.04), had lower LVEF% (p<0.0001), higher CHA2DS2-VASc score (p<0.0001), and higher rate of coronary artery disease (CAD) (p=0.03)	Echocardiography before electric cardioversion identifies clear LAT/SEC in more than a third of AF patients, independently by their anticoagulation regimen. LAT/SEC rates increase with decrement in LVEF%. Increment in CHA2DS2-VASc score increases SEC risk.		

Discussion

The hypothesis that AF must persist for more than two to three days before a LAT form has gained widespread acceptance, even though the mechanism of LAT formation and subsequent embolization is complex and little understood [19]. Still, later on, it was proved false as there were many cases of thrombus formation with AF in less than two days. According to the FibStroke study, 21% of the patients who developed an ischemic stroke or TIA after cardioversion had a CHA2DS2-VASc (congestive heart failure, hypertension, age ≥75 (doubled), diabetes, stroke (doubled), vascular disease, age 65 to 74 and sex category (female)) score < 2 [25]. AF is associated with an increased risk of heart failure, thromboembolism, and death [9]. DCC is an effective procedure to restore sinus rhythm. It is widely used in patients with persistent AF patients undergoing DCC in moderate to relatively high-risk categories based on the CHA2DS2-VASc scoring scheme [6,8]. Yarmohammadi et al. reported no clear trend in the incidence of LAA thrombus, stroke, or embolic events across patients with different CHA2DS2-VASc scores [6]. TEE allows accurate detection of LAT.

Moreover, recent studies using TEE have shown a state of atrial stunning immediately after cardioversion, a thrombogenic milieu in which new thrombus formation and increased or de novo appearance of LA spontaneous echocardiographic contrast (SEC) have been observed [25]. Over the past 10 years, trends display that the application of TEE-guided DCC has consistently grown and that more DCC procedures are done in the outpatient setting. Given TEE's high LAT or sludge detection rate, TEE-guided DCC remains an essential part of AF management [14]. In AF patients undergoing cardioversion, contrast-enhanced TEE images are more interpretable, help exclude atrial thrombi, and may result in a decreased rate of embolic adverse events [12]. TEE helps to detect LAA thrombus and defer the cardioversion in selected patients. TEE before cardioversion does not eliminate the risk of embolism after cardioversion because of atrial stasis and new thrombosis [8]. Cardioversion should be performed inadequately in anticoagulated patients even when TEE shows no LAT. Hence, TEE is not an alternative to anticoagulant therapy [16].

Cardioversion briefly impairs the left ventricle contraction and creates a stasis which can be a thrombogenic milieu; hence, full anticoagulant therapy should be administered before undergoing cardioversion [7]. Electrical cardioversion disrupts the function of the LAA and could increase the risk of thrombus formation [7,26]. In patients with unknown or prolonged duration AF who are not receiving long-term anticoagulation, atrial thrombi are detected by TEE in only a small minority of patients. It suggests that if TEE excludes the thrombi, early cardioversion can be performed safely without needing prolonged oral anticoagulation before the procedure [10]. It is possible to selectively screen patients to identify those at low risk for developing thrombi subsequent to negative results on initial TEE, especially if patients are in sinus rhythm [11]. Sludge within the LAA is independently associated with subsequent thromboembolic events in patients with AF [27]. Decreased LAA emptying flow velocity is related to recurrent AF even after successful cardioversion; hence, real-time measurement of emptying velocity by TEE can help decide treatment in patients with AF [15]. The presence of left ventricular dysfunction or mitral stenosis in patients with acute AF or left atrial enlargement in patients with chronic AF is not a reliable predictor of developing a LAT [19]. Improved and automated image interpretation will allow us to understand the LA and LAA anatomy better and possibly detect specific anatomical features and early signs correlated to LAT and SEC occurrence [24]. TEE-guided cardioversion provided a safe, effective, and noninvasive means of establishing atrioventricular synchrony and improving ventricular systolic function while concomitantly allowing surveillance of intracardiac thrombi [28]. Left atrial spontaneous echo contrast was the only independent positive predictor of subsequent thromboembolic events, including stroke, transient ischemic attack, and peripheral embolism [29]. Most post-cardioversion strokes occur in patients not using oral anticoagulation before cardioversion of acute AF [30]. In previous studies, the risk of stroke after cardioversion guided by TEE has been 0.8% [31,32].

## Conclusions

There has been a significant increase in the utilization of TEE-guided DCC mainly because it allows for the detection of atrial thrombi before cardioversion, which is important for risk stratification, especially in outpatient settings. The detection of a thrombus in the left atrium is also correlated with an increased risk of future thromboembolic events in patients with AF. Furthermore, atrial stunning detected on TEE after cardioversion is a significant risk factor for future thromboembolic events; however, further evidence is required to support this claim. Additionally, therapeutic anticoagulation is needed during and after cardioversion even if no atrial thrombus is detected on TEE. Finally, based on currently available data, it is recommended that TEE-guided cardioversion be performed, especially in outpatient settings.
